# Phylodynamic analysis of Salmonella Enteritidis ST183 in Aotearoa New Zealand finds no evidence for introduction via European hedgehogs (Erinaceus europaeus)

**DOI:** 10.1099/mgen.0.001677

**Published:** 2026-03-18

**Authors:** Hugo Strydom, Shevaun Paine, David Welch, Jackie Wright, Collette Bromhead, Chris N. Niebuhr, Ernest Williams, Jennie Fischer, Laura Uelze, Sandra Simon, Michael Pietsch, Becki Lawson, Marie Anne Chattaway, Sarah Jefferies, Joep de Ligt, Nigel French

**Affiliations:** 1 Health Security, New Zealand Institute for Public Health and Forensic Science, Wellington, New Zealand; 2 Tāwharau Ora | School of Veterinary Science, Massey University, Palmerston North, New Zealand; 3 School of Computer Science, University of Auckland, Auckland, New Zealand; 4 School of Health Sciences, Massey University, Wellington, New Zealand; 5 Manaaki Whenua – Landcare Research Group, Bioeconomy Science Institute, Lincoln, New Zealand; 6 Food Microbiology, Host-Pathogen-Interaction/German National Reference Laboratory for Salmonella, German Federal Institute for Risk Assessment, Berlin, Germany; 7 Unit of Enteropathogenic Bacteria and Legionella/National Reference Centre for Salmonella and Other Bacterial Enteric Pathogens, Robert Koch Institute, Wernigerode, Germany; 8 Institute of Zoology, Zoological Society of London, Regent’s Park, London, England, UK; 9 Gastrointestinal Bacteria Reference Unit, United Kingdom Health Security Agency, London, England, UK; 10 NIHR HPRU in Genomics and Data Enabling, University of Warwick, Coventry, England, UK; 11 New Zealand Food Safety Science and Research Centre, Massey University, Palmerston North, New Zealand

**Keywords:** genomic epidemiology, New Zealand, wildlife

## Abstract

*Salmonella enterica* subsp. *enterica* serovar Enteritidis (*S*. Enteritidis) is the second most common serovar causing human salmonellosis in Aotearoa New Zealand (NZ). Sequence type (ST)183, which includes phage types 9a and 11, was the second most frequently isolated *S*. Enteritidis strain from human cases between 2020 and 2023. This ST is considered endemic in NZ as well as in mainland Europe and Great Britain, where the European hedgehog (*Erinaceus europaeus*) is a recognized wildlife reservoir. Hedgehogs were introduced to NZ in the late 19th century; however, their role in the ecology of ST183 in NZ has not been formally evaluated. The aim of this study was to investigate whether hedgehogs act as a reservoir for *S*. Enteritidis ST183 in NZ and to assess the evolutionary history and epidemiology of this strain across human, animal and environmental contexts. We analysed human, animal and environmental ST183 isolates, including hedgehog carcasses opportunistically sampled in NZ, using Bayesian phylogenetic methods integrated with national epidemiological data. Although *S*. Enteritidis ST183 was isolated from three of 45 hedgehog carcasses during our study, consistent with recent prior detections of ST183 in NZ hedgehogs, Bayesian phylogenetic analysis supports a most recent common ancestor for currently circulating ST183 strains in the late 20th century, ~100 years after the introduction of hedgehogs into NZ, providing no evidence that the strain was introduced concomitantly with hedgehogs. Epidemiological analysis revealed that, unlike in Europe, ST183 infections in NZ are more common in people aged over 60 years compared with non-ST183 *S*. Enteritidis infections, with rural residence and contact with farm animals identified as key risk factors. Together, these findings suggest that *S*. Enteritidis ST183 is established within the NZ rural environment, with evidence of interspecies transmission. While hedgehogs may contribute to the maintenance of ST183, they are unlikely to represent the original source of introduction, indicating a complex, multi-host ecology.

**Impact Statement**

This study offers valuable insight into the phylodynamics of *Salmonella* Enteritidis ST183 in New Zealand (NZ). It shows that the ST183 bacterial population endemic in NZ is separate from ST183 populations in Great Britain and Germany. We estimated the most recent ancestor for NZ ST183 and determined that it did not coincide with the introduction of European hedgehogs to NZ. However, we confirmed that ST183 is common in the NZ environment and that European hedgehogs are one of several plausible animal hosts. By integrating disease modelling with genomic and epidemiological data, this study provides public health investigators with a better understanding of zoonotic transmission, enabling more targeted interventions to reduce the burden of disease. In addition, the study contributes additional *S*. Enteritidis genomic data to the public arena.

## Data Summary

This study reports previously unpublished sequence data from *S*. Enteritidis ST183 clinical human isolates submitted to the New Zealand Institute for Public Health and Forensic Science (PHF Science). Furthermore, it includes sequences from isolates obtained from freshwater sources, domestic animals, hedgehogs and food sources in New Zealand (PRJNA720150). For global comparison, we included *S*. Enteritidis ST183 short-read sequences previously isolated and sequenced in Germany and published under the NCBI BioProject PRJEB31846 and the GenoSalmSurv project. Previously unpublished short-read sequences from clinical human and wildlife isolates were provided by the Robert Koch Institute (RKI) (PRJEB100830) and the German Federal Institute for Risk Assessment (BfR) (PRJNA937468), respectively. Short-read sequences from hedgehog isolates by the Zoological Society of London as part of the Garden Wildlife Health project and clinical human isolates sampled and sequenced in Great Britain by the UK Health Security Agency published under NCBI BioProject PRJNA248792 were also part of this study.

The authors confirm that all supporting data, code and protocols have been provided within the article or through supplementary data files.

## Introduction

*Salmonella enterica* is an important global cause of gastroenteritis in humans and other animals [1]. Food, contact with companion animals, livestock and wildlife and to a lesser extent person-to-person contact have previously been identified as sources of human infection [2]. *S. enterica* subsp. *enterica* serovar Enteritidis (henceforth *S*. Enteritidis) is the second most identified serovar in Aotearoa New Zealand (NZ), with *S*. Typhimurium being the most common [3]. *S*. Enteritidis can be subdivided into 60 phage types (PTs) based on lysogenicity of strains against a panel of bacteriophages as described by Ward *et al.* [4]. However, phage typing alone does not allow for relatedness to be measured on a molecular level. Nauerby *et al.* concluded that *S*. Enteritidis PT9a and PT11 belong to the same clonal lineage [5]. Seven-gene multilocus sequence typing (MLST) sequence type (ST)183 is endemic in mainland Europe and Great Britain (GB) and includes PT11, PT9a and PT66 [5,7]. While *S*. Enteritidis PT66 has not been detected in NZ, PT11 and PT9a have been isolated in NZ since phage typing was introduced as a routine subtyping method for *S*. Enteritidis in the 1990s. Following a period of validation where genomic and phenotypic methods were used for a selection of isolates, sequence typing replaced phage typing in November 2019 as the in-use subtyping method by the national Enteric Reference Laboratory (ERL) at the NZ Institute for Public Health and Forensic (PHF) Science.

ST183 is also considered endemic in NZ, and from January 2016 to July 2021, 87 human clinical isolates referred for epidemiological typing according to NZ Ministry of Health requirements [8] were confirmed as ST183. In February 2021, an outbreak of ST183 involving 28 people was identified [9]. Seven patients were hospitalized, and cases were reported across the South Island (*n*=25) and lower North Island (*n*=3), indicating geographic clustering as only 24% of the NZ population reside in the South Island [10]. SNP analysis showed isolates were near clonal, indicating a potential common source. Packaged alfalfa and radish sprouts, which are typically grown in NZ and not imported, were identified as the suspected source, based on epidemiological and whole-genome sequencing linkage [11]. A much larger nationwide outbreak of *S*. Enteritidis ST11 occurred from 2019 to 2022 associated with poultry [12]; subsequently, ST183 was the second most common *S*. Enteritidis ST isolated between 2020 and 2023.

In mainland Europe and GB, ST183 has been associated with the European hedgehog (*Erinaceus europaeus*, henceforth hedgehog) [5,7,13]. *S*. Enteritidis in hedgehogs was first described by Buxton and Field [16,17]. A more recent study examining the impact on both hedgehogs and public health in GB found that the hedgehog was the non-human species from which PT11 (ST183) was most frequently isolated, with only a small number of detections in companion animals, livestock and wild birds in recent decades, which represented a minority of PTs from these hosts [7]. This study found no strong association between phylogeny and host species, suggesting interspecies transmissions. In addition, this study suggested hedgehogs are the principal reservoir for PT11 (ST183) infection [7]. The hedgehog is listed as a species of conservation concern in GB [18,19] and several other European countries [20], and it is the most frequently admitted mammal to wildlife rehabilitation centres in GB [21]. In GB, PT11 (ST183) was significantly more likely to be reported in children aged 0–4 compared to *S*. Enteritidis ST11, the ST that causes the majority of *S*. Enteritidis infections in England and Wales [7]. In addition, infection due to PT11 (ST183) in GB was significantly more likely for people living in rural rather than urban areas [7]. However, between January 2006 and December 2015 in England and Wales, PT11 (ST183) accounted for only 1.6% of *S*. Enteritidis isolated from human clinical cases [7].

Another study investigating the genetic diversity of *Salmonella* in wildlife found that ST183 was the only ST isolated from hedgehogs in Germany [15]. This serovar was also found in several other wildlife hosts such as red fox, deer, wild birds and wild boar. In addition, phylogenetic analysis supported frequent interspecies transmission events including wild, farmed and companion animals. The study concluded that ST183 is host-adapted, but not strictly host-restricted to hedgehogs and that hedgehogs were the principal ST183 reservoir in Germany [15].

In NZ, ST183 has been isolated from a variety of farmed animals, companion animals, hedgehogs, food sources, animal feed and the environment [22]. A NZ study in 1995 reported a significant regional difference with *S*. Enteritidis PT9a more prevalent in lymph node samples from hedgehogs from the Wairarapa region (lower North Island) than from North Canterbury and Otago regions (both South Island) [23]. Unfortunately, none of these isolates were available for further analysis at the time of our study.

Introduced mammalian predators like hedgehogs severely impact NZ’s native biodiversity [24] and are a focus of many trapping programmes and control efforts. The 1861 Animal Acclimatisation Act encouraged the introduction of animals to NZ which would ‘contribute to the pleasure and profit of the inhabitants’ [25]. The first introduction of hedgehogs to the South Island of NZ was planned by the Canterbury Acclimatisation Society in 1869, using animals sourced from GB, to control garden pests in domestic gardens [26,27]. However, only two hedgehogs survived the journey to NZ and were likely never released [28]. The first reported South Island release occurred in Canterbury in 1881 [28]. However, escaped pet hedgehogs were reported in the North Island by 1880 in Wellington and Auckland [25,28]. Bolfíková *et al.* identified a Palmerston North population as a bridgehead population that dominated the founding gene pool of NZ’s current hedgehog lineage [29]. Favourable environmental conditions and hedgehogs’ synanthropic nature resulted in population densities exceeding those in GB [29]. Between 1930 and 1939, hedgehog numbers increased dramatically on both islands to the detriment of wild bird populations [25]. NZ has no native terrestrial mammals except for two species of bats which resulted in the evolution of an abundance of flightless and ground-nesting birds particularly vulnerable to predation [30]. By 1939, responding to game bird hunters’ complaints, the Ministry of Internal Affairs and Acclimatisation Societies funded ‘vermin’ eradication programmes targeting swamp harriers (*Circus approximans*), stoats (*Mustela erminea*), ferrets (*Mustela furo*), weasels (*Mustela nivalis*) and hedgehogs [25].

Using Bayesian temporal analysis, we investigated whether the ancestral lineage of the current circulating ST183 strain in NZ is associated with the introduction of hedgehogs in the late 19th century. In addition, by comparing risk factors associated with ST183 infection in NZ with those experienced in mainland Europe and GB, we evaluated the feasibility of hedgehogs being a reservoir of ST183 and a source of human infection in NZ.

## Methods

### Epidemiological analyses of *S*. Enteritidis cases

Epidemiological analyses were conducted on 367 human cases (2020–2023 inclusive) from whom *S*. Enteritidis was isolated and for which the ST was determined using whole-genome sequencing as routine epidemiological typing for public health surveillance via an ISO 15189-accredited process. Demographic and risk factor information is routinely collected for salmonellosis cases in NZ for public health surveillance purposes [31]. These data were submitted by local public health services to the national notifiable disease database, EpiSurv. For the period 2020 to 2023, age was grouped into infants 0–4 years, older children 5–19 years and adults 20–29, 30–39, 40–49, 50–59 and 60 years and older (60+). Age groups and rural residence for ST183 and non-ST183 cases were measured against population denominator data in cases per million person years at risk, and then, the median rate ratio, 95% confidence interval (95% CI) and mid-*P*-values were calculated relative to the largest ten year age group (30–39) or urban residence, respectively, using the ‘epitools’ package in R v4.4.1. Residences were classified as urban or rural using the Stats NZ’s (NZ’s official data agency) Urban–Rural 2023 (UR2023) classification, which defines urban areas based on population size, density and the extent of built-up land and functional connectivity, with all remaining areas classified as rural [32,33]. The incidence rates per age group were visualized using ‘ggplot2’ [34]. Exposure proportions were calculated for ST183 and non-ST183 cases individually compared to ST183 and non-ST183 combined. Pearson’s chi-squared test with Yates’ continuity correction and Fisher’s exact test were performed using R v4.4.1. In addition, in a case–case study, the odds ratio of exposures of ST183 cases relative to the exposures of non-ST183 cases was estimated. A *P*-value less than 0.05 was considered statistically significant. The incubation period was defined as the time between exposure to the pathogen and the onset of clinical symptoms.

### Sampling from hedgehogs in NZ

This study sampled 45 hedgehog carcasses from both the North and South Island of NZ, at multiple locations within Canterbury, Otago and Waikato. Samples were taken from hedgehog carcasses collected from various routine pest management efforts and freshly collected roadkill in NZ, during 2023 and 2024. Only carcasses with date and location information and no clear sign of decomposition were sampled. Hedgehog carcasses were frozen at −20 °C prior to dissection when 5 g of faeces was collected from the large intestinal tract and 2 g of liver was collected, transferred to Cary–Blair tubes and sent to the ERL at PHF Science for further analysis. All media were sourced from Fort Richard Laboratories, Auckland, NZ. For each sample, faeces and liver were homogenized as a composite and spread on a Hektoen enteric (HE) agar and xylose lysine deoxycholate (XLD) agar plates. Two drops (~0.1 ml) of homogenized sample were dropped in Rappaport–Vassiliadis soya broth and incubated for 24 h at 37 °C, followed by plating onto HE and XLD agar. Typical colonies were selected from HE and XLD agars and inoculated onto tryptic soy agar (TSA). In addition, three ST183 isolates from hedgehog samples in NZ previously submitted for animal health surveillance were also included in this study.

### Isolate and genome selection for sequencing

A total of 149 NZ ST183 isolates were randomly selected from the available archive for inclusion in this study, as outlined below. Short-read sequence data for ST183 isolates were obtained from multiple sources. These included *S*. Enteritidis PT9a and PT11 isolates from human clinical cases submitted to ERL for public health surveillance between June 2013 and November 2019 inclusive [8]. Isolates from this period were stratified by year of collection and randomly selected using a random number generator (https://www.calculator.net/random-number-generator.htm), then sequenced and included in this study.

Additional ST183 short-read sequences were included from human clinical isolates submitted to ERL between November 2019 and September 2022 inclusive, which were routinely sequenced as part of public health surveillance, as well as from hedgehogs (*n*=6: three isolated from hedgehog carcasses sampled during this study and three historical isolates, sampled in 2002 and 2006), freshwater sources (*n*=8), domestic animals (three cats, one dog and one sheep) and food sources (packaged alfalfa and radish sprouts, *n*=1). Domestic animal isolates and three of the hedgehog isolates were submitted to ERL for diagnostic and surveillance purposes. Freshwater isolate genomic reads were from previous environmental studies [35,36]; and the food isolate originated from an outbreak investigation.

For all NZ isolates, bacteria were cultivated on TSA at 37 °C for 18 h. Serovar identification was performed by slide agglutination using O- and H-specific antisera (Sifin Diagnostics) according to the White–Kauffmann–Le Minor scheme published by the World Health Organization (WHO) [37].

For global comparisons, we included 27 short-read sequences from Germany previously isolated and sequenced, published under the NCBI BioProject PRJEB31846 [15] and the GenoSalmSurv project [38]. Additional short-read sequences were provided by the German Federal Institute for Risk Assessment (BfR) from wildlife sources and by the Robert Koch Institute (RKI), which provided sequences from clinical human isolates not previously published. In total, 152 German short-read sequences were included, comprising isolates from humans (*n*=56), hedgehogs (*n*=49), other wild animals including wild birds (*n*=22), companion animals (*n*=10), poultry (*n*=7), livestock (*n*=4), herbs (*n*=2) and unknown sources (*n*=2).

In addition, 23 short-read sequences from hedgehog isolates obtained through wildlife surveillance by the Zoological Society of London were included, along with 124 short-read sequences from human clinical isolates sampled and sequenced by the UK Health Security Agency in GB, published under NCBI BioProject PRJNA248792 [7,39]. Further detail is available in Tables 1 and S1 (available in the online Supplementary Material).

**Table 1.** Genomes included in this study from *Salmonella* Enteritidis ST183 isolated from a variety of host species in Germany, GB and NZ

NZ		Germany		GB	
Human	129	Human	56	Human	124
Hedgehog	6	Hedgehog	49	Hedgehog	23
Cat	3	Other wildlife	22		
Dog	1	Cat	5		
Freshwater	8	Dog	5		
Sheep	1	Poultry	7		
Alfalfa sprouts	1	Herbs	2		
		Sheep	1		
		Pig	1		
		Goat	1		
		Unknown	3		
Total	149		152		147

Additional detail available in Table S1.

All *S*. Enteritidis PT66 sequences found in the GB dataset [7] were excluded from the analysis because this PT is not known to be present in NZ and was not included in the German datasets.

### Whole-genome sequencing

Genomic DNA extraction, library preparation and whole-genome sequencing of NZ isolates were performed by the sequencing facility at PHF Science. DNA extractions were performed using a Chemagic™ 360 platform and the Chemagic Viral DNA/RNA 300 Kit H96 (CMG-1033-EX) (Revvity). DNA library preparation was generated using the Nextera XT (Illumina, San Diego, CA, USA) or SeqWell purePlex™ (SeqWell, MA, USA) DNA Library Prep Kit, and sequencing was performed using an Illumina MiSeq, Nextseq 550 or Nextseq 2000 platform (Illumina), producing 2×150 bp paired-end reads. Previously unpublished sequence data from BfR were attained as previously described [15]. Genomic DNA was extracted from clinical isolates at the RKI using either the GenEluteT Bacterial Genomic DNA Kit from Sigma-Aldrich (St Louis, MO, USA) or by mechanical disruption using 425–600 micron glass beads (Sigma-Aldrich) in a TissueLyser II bead mill (Qiagen, Hilden, Germany). Library preparation (Illumina Nextera XT Library Preparation Kit), short-read sequencing (2×300 bp MiSeq, 2×250 bp NextSeq 500 or 2×150 bp NextSeq 2000) and subsequent quality control of raw sequence data were performed at the Sequencing Core Facility within the Genome Competence Centre at RKI.

### Genome assembly

Genome sequence trimming, *de novo* assembly and quality assessments were performed using the AQUAMIS pipeline v1.3.7 [40] on all reads. The AQUAMIS pipeline implements fastp v0.23.2 [41] for trimming, Shovill v1.1.0 [42] for assembly, QUAST v5.0.2 for quality assessment and ConFindr v0.7.4 [43] for contamination detection. Taxonomic classifications were confirmed using Kraken v2.1.2 [44]. Sequences were excluded from further analysis based on the following criteria: coverage depth below 30; bacterial inter- or intra-species contamination consisting of 8 or more alleles of core, single-copy genes; total length exceeding 5.1 Mb; more than 400 contigs; and a Q30 base frequency of less than 0.75. Genomic reads from a total of 448 isolates were included for further analysis.

### *Salmonella* pathogenicity islands and genotypic antimicrobial resistance

Characterization of ST183 genetic determinants was performed using draft genome assemblies using the BakCharak v3.0.4 [45] pipeline. This pipeline implements ABRicate v1.0.1 [46] to detect antimicrobial resistance (AMR) genes using NCBI AMRFinderPlus v3.11.11 [47,48], plasmid prediction using Platon v1.6 [49], plasmid compatibility groups from the PlasmidFinder database [50] and virulence genes from the Virulence Factors Database (VFDB) [51]. Furthermore, this pipeline implements sistr cmd v1.1.1 [52,53], mlst v2.23.0 [54] and PubMLST schemes (https://pubmlst.org/) for seven-gene MLST typing.

### Phenotypic AMR

Six NZ human clinical isolates included in our study were also randomly selected as per standard protocol for *S*. Enteritidis submitted to PHF Science for public health purposes, and phenotypic antimicrobial susceptibility was determined by the European Committee on Antimicrobial Susceptibility (EUCAST) disc diffusion method [55]. The test panel included the following antimicrobial agents: amoxicillin–clavulanic acid, ampicillin, chloramphenicol, co-trimoxazole, cefotaxime, ceftazidime, gentamicin, meropenem, pefloxacin and tetracycline. Results were interpreted using EUCAST breakpoints current for the year the isolate was received at PHF Science [56] except for tetracycline for which zone diameters were interpreted using Clinical and Laboratory Standards Institute (CLSI) breakpoints, current for the year the isolate was received at PHF Science [57,58].

### Analysis of SNPs

Genomic sequences for isolates sampled from NZ (*n*=149), Germany (*n*=152) and GB (*n*=147) were included in the phylogenetic analyses. SNP calling and filtering were performed using snippySnake v1.2.3 [59] that uses snippy v4.4.3 [60] to align sequences against the *S*. Enteritidis reference genome NZ_CP025554.1 (ATCC BAA-708) and to identify core SNPs. SNP-dist v0.6.3 [61] was used to generate a SNP distance matrix. Recombinant regions were detected and removed using Gubbins v3.2.1 [62]. Following alignment, the reference sequence was manually deleted, and SNPs were extracted using SNP-sites v.2.4.1 [63], resulting in an alignment consisting of 4,126 SNPs and 1,395 parsimony-informative SNP sites. A maximum likelihood phylogenetic tree was inferred using RaxML-NG v1.2.2 [64]. The phylogenetic tree was visualized using Interactive Tree of Life (iTOL) v6.5.8 [65]. Statistical hierarchical clustering was performed using RhierBAPs v1.0.1 [66], estimating up to three levels with the number of populations set at four.

### Temporal phylogenetic reconstruction

For temporal phylogenetic reconstruction, all available sequences were stratified into country of origin and human versus non-human source. Twenty human and 20 non-human sequences were randomly selected from each of the German and GB sequence datasets. For the NZ samples, 25 human isolates and 15 non-human isolates (including 6 hedgehogs) were included in the study. Only one representative human isolate associated with the 2021 outbreak was included. Further detail is available in the supplementary material (Table S1). To determine the temporal signal, a maximum likelihood phylogenetic tree based on SNP distance was generated using RaxML-NG v1.2.2 [64] and a linear regression of root-to-tip genetic distance against sampling times was constructed using TempEST v1.5.3 [67]. Following alignment, recombinant regions were identified and removed using Gubbins v3.2.1 [62]. The reference sequence was manually deleted, and SNPs were extracted using SNP-sites v.2.4.1 [63], resulting in an alignment consisting of 2002 SNP sites with 485 parsimony informative sites. Bayesian analyses to determine the time to the most recent common ancestor (tMRCA) and the substitution rate for the total ST183 bacterial population (NZ, GB and Germany), and the tMRCA and population dynamics for the NZ-only ST183 bacterial population were performed using Bayesian Evolutionary Analysis by Sampling Trees (BEAST2) v2.7.4 [68] in triplicate with a different seed used in each iteration. Hasegawa–Kishino–Yano (HKY) [69], the general time-reversible [70] and bModelTest [71] substitution models were compared; however, they showed no significant differences between estimated parameters of interest (data not shown). Therefore, the HKY model was chosen for simplicity and computational speed. Every attempt to use the optimized relaxed clock failed to converge, so a strict clock was used instead. Coalescent constant, coalescent exponential and the non-parametric Bayesian Integrated Coalescent Epoch PlotS (BICEPS) [72] tree priors were compared using Path-sampling [73,75] and the BICEPS model was selected. Thus, the BICEPS tree prior, HKY substitution model and a strict clock prior were chosen to estimate the tMRCA and substitution rate.

The clock rate prior was based on a previously published estimate [76] with a log normal distribution, a mean of 2.2E−7 and a sd of 0.5. The prior for the BICEPS effective population size had a gamma (6.4) distribution. The model was fit using Markov chain Monte Carlo (MCMC) run for 10 million iterations sampled every 1000 iterations. For the estimation of the tMRCA and population dynamics, the NZ subset was also run independently with the same conditions as described above except the prior for the BICEPS effective population size had a gamma (4,2) distribution and the model was fit using MCMC run for 30 million iterations sampled every 3,000 iterations. Posterior estimates of various parameters sampled by the Markov chain were summarized using Tracer v1.7.2. Qualitative analyses of the resulting sets of trees were performed using Densitree v3.0.2 [77]. Maximum clade credibility trees were summarized using TreeAnnotator v2.7.4 [78] and visualized using FigTree 1.4.4 and iTOL v6.5.8 [65]. The substitution rate, as well as the tMRCA distributions for the Germany and GB populations, was inferred based on the maximum clade credibility tree summarized based on mean values. The tMRCA distribution for NZ was analysed using Tracer.

## Results

### Epidemiology of notified human cases of ST183 in NZ

**Fig. 1.**
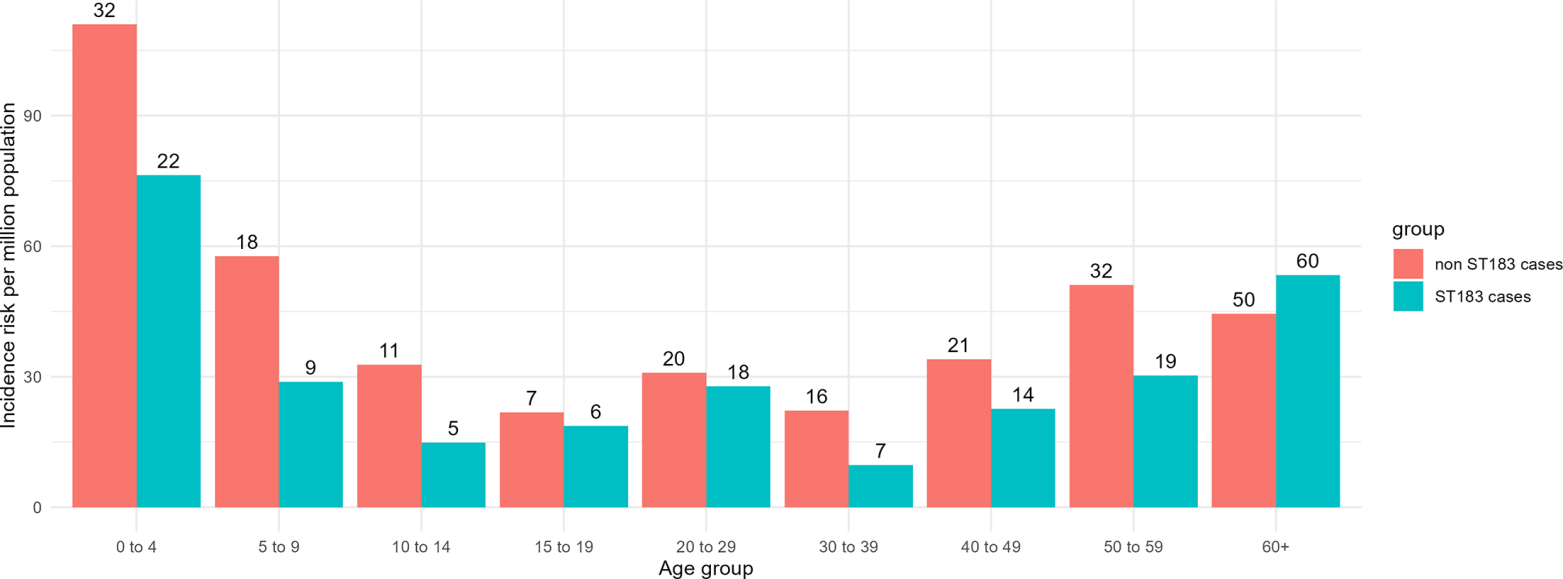
The incidence rate of *Salmonella* Enteritidis ST183 and non-ST183 in NZ with the number of notified human cases (2020–2023) above the bars. The green bars represent the incidence risk for ST183 human cases, the red bars represent the incidence risk for non-ST183 human cases.

From January 2020 until December 2023, *S*. Enteritidis ST 183 was the second most frequently notified *S*. Enteritidis ST in NZ (160/367, 44%; Table S2). The sample source was recorded for 154 out of 160 ST183 samples received. Of these 148 (96%) were from faecal samples, implying that the presenting symptoms were mainly gastrointestinal in nature. The most notified *S*. Enteritidis ST was ST11 (193/367, 53%) (Table S2) due in part to a prolonged outbreak of ST11 between 2019 and 2023 [79]. We compared the age distribution of notified ST183 cases and non-ST183 cases. An analysis of estimated incidence rates relative (RR) to the largest 10-year age group (30–39) showed that the rates for both the young (0–4) and the old (60+) age groups were significantly higher than the reference group for both ST183 and non-ST183 cases [ST183 – 0–4: RR =7.7, 95% CI (3.4, 19.8), *P* <0.000001; 60+: RR = 5.4, 95% CI (2.6, 13), *P*< 0.000001; non- ST183 – 0–4: RR =5, 95% CI (2.8, 9.3), *P* <0.000001; 60+: RR =2, 95% CI (1.2, 3.6), *P* = 0.012] (Fig. 1, Table S3). When comparing the incidence rate of the age group 0–4 relative to all the other ages combined, we found that the relative rate for both ST183 [RR = 2.6, 95% CI (1.6, 4), *P* = 0.0002] and non-ST183 [RR = 3, 95% CI (2.0, 4.3), *P* = 0.000001] was significantly higher, with overlapping 95% CI. However, when comparing the incidence rate of the age group 60+ relative to all the other ages combined, we found the relative rate was significantly higher for ST183 [RR = 2.1, 95% CI (1.5, 2.8), *P* = 0.00002] but not for the non-ST183 [RR = 1.1, 95% CI (0.8, 1.5), *P* = 0.570] (Table S3).

In NZ, 15.6% of the human population live in rural areas [32,33]. The proportion of ST183 cases that lived in rural areas (29.7%, 43/145) was higher than the proportion of non-ST183 that lived in rural areas (15.5%, 30/193). When comparing the incidence rate of cases that live in rural areas relative to cases that live in urban areas, we found the relative rate was significantly higher in rural dwellers for ST183 [RR = 2.3, 95% CI (1.6, 3.2), *P* = 0.00002] but not for non-ST183 [RR = 1, 95% CI (0.7, 1.5), *P* = 1.00] (Table S4).

The proportion of ST183 cases that reported contact with farm animals (26.8%, 33/123) was significantly higher than for non-ST183 cases (14.0%, 19/136), (*χ*^2^=5.9, df=1, *P*-value=0.013) (Table S5). None of the cases reported contact with hedgehogs, although information about contact with hedgehogs or other wild animals is not routinely collected by public health services. The proportion of ST183 cases that reported exposure to recreational water (12.2%, 15/123) was significantly lower than for non-ST183 cases (23.6%, 33/140) (*χ*^2^=4.9, df=1, *P*-value=0.02) (Table S5). No ST183 cases reported recent international travel, while 31.3% (57/182) of non-ST183 cases reported international travel during their incubation period (*χ*^2^=50.1, df=1, *P*-value<0.000001).

### Genotypic and phenotypic characterization of isolates from NZ, Germany and GB

Six randomly selected NZ clinical human ST183 isolates from this study were phenotypically susceptible to all the antimicrobials tested including the aminoglycoside gentamicin (Table S6). The AMR genes coding for the *Salmonella*-specific resistance–nodulation–division (RND) efflux pump MsdAB (*msdA* and *msdB*) were detected in 99.3% of the 448 genomes tested. The remainder (*n*=3) contained only *msdB*. The gold/copper-translocating P-type ATPase GolT and the Au(I) sensory transcriptional regulator GolS were detected for all isolates tested. The plasmid incompatibility group IncFII(S)_1 was predicted for all genomes tested. Three genomes (0.7%) contained replicons for both IncFII(S)_1 and IncI1_1_Alpha; one genome (0.2%) contained markers for both IncFII(S)_1 and IncI_Gamma_1 plasmid; and one genome (0.2%) contained markers for both IncFII(S)_1 and ColRNAI_1.

### Phylogenetic study of combined NZ, German and GB *S*. Enteritidis ST183 populations

**Fig. 2.**
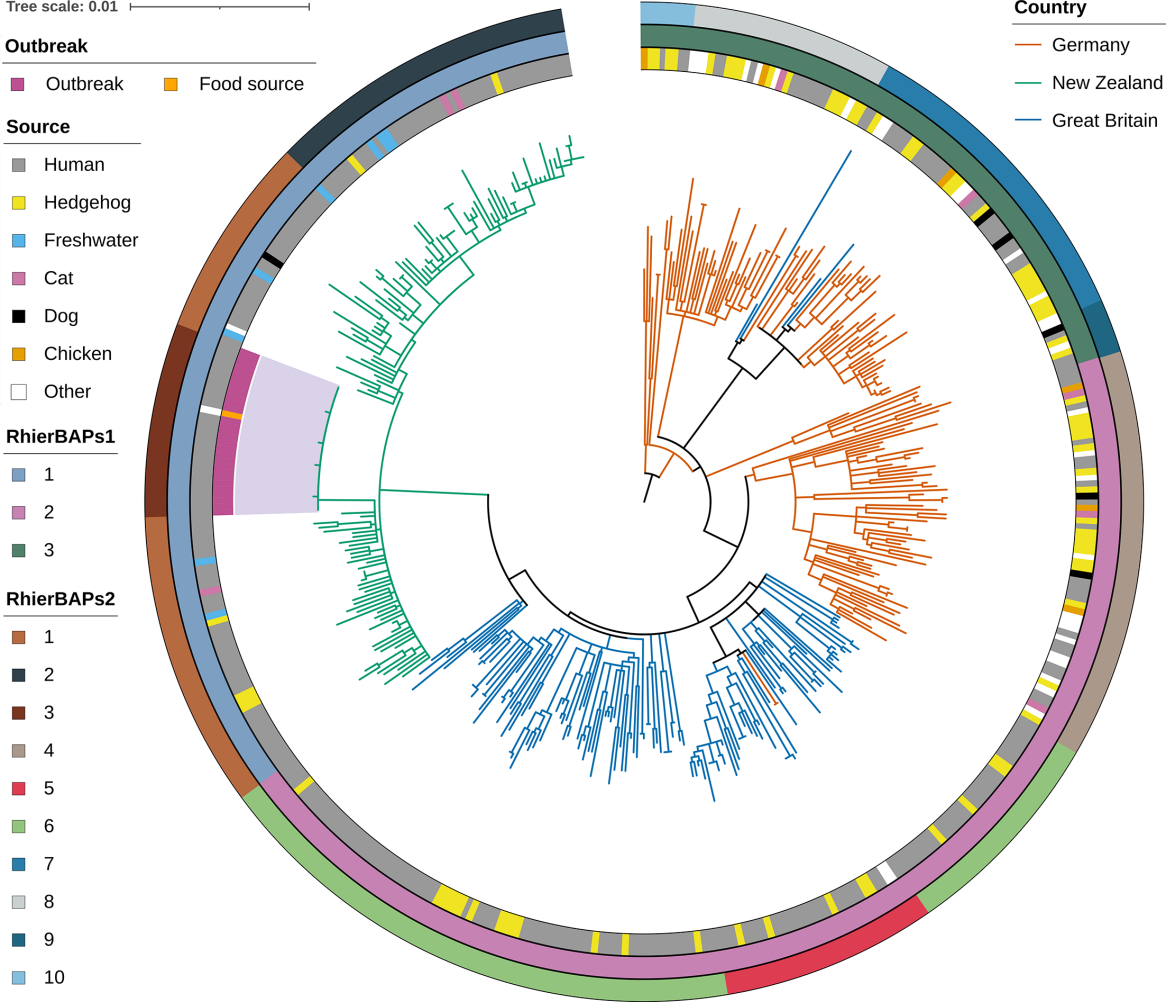
Maximum likelihood phylogenetic tree based on 4126 core SNPs of combined populations (*n*=448) of *Salmonella* Enteritidis ST183 from Germany, GB and NZ. The tree was manually rooted to the reference genome (NZ_CP025554.1) and visualized using iTOL. Branches are coloured according to the country that the leaf tip was sampled from: Germany, orange; GB, blue; and NZ, green. The innermost complete colour ring shows the source from which ST183 was isolated. The middle and the outer complete coloured rings show clades as determined using RhierBAPs level 1 and level 2, respectively. The clade containing the 2021 NZ outbreak is shaded purple, with isolates from human patients in deep pink and food source in yellow.

*S*. Enteritidis was isolated from 3 out of 45 hedgehogs sampled. All isolates were ST183. Hierarchical clustering (level 2 in RhierBAPs) of genomes from 448 isolates sampled from NZ, Germany and GB showed multiple distinct clusters within each country of origin (Fig. 2) apart from six isolates from Germany and GB which clustered within the clade of the other country of origin, respectively. There was no apparent association between phylogeny and host species. The 28 isolates belonging to the 2021 outbreak in NZ clustered with the suspected food source (0 or 1 core SNP). All genomes tested were within 137 core SNPs from each other. All NZ genomes were within 41 core SNPs from each other and the closest non-NZ genome to the NZ clade was isolated from a person in GB in 2016 which was 40 core SNPs from the nearest NZ isolate.

### Bayesian phylogenetic analysis

**Fig. 3.**
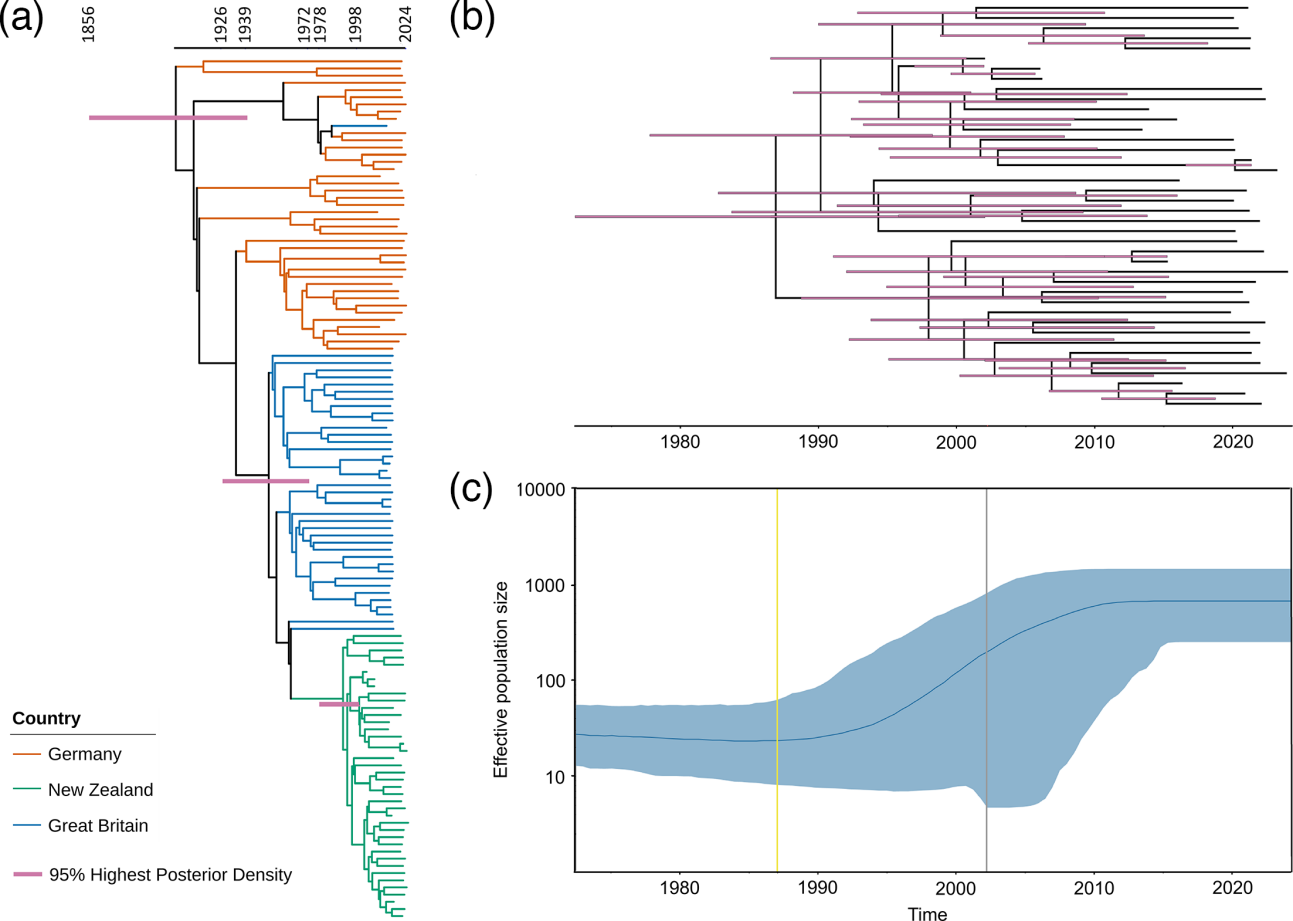
(a) Maximum clade credibility tree of *Salmonella* Enteritidis ST183 populations generated using BICEPS. Tree branches are coloured according to the country in which the isolate was sampled from the following: Germany, orange; GB, blue; and NZ, green. (b) Maximum clade credibility tree generated for NZ isolates only using BICEPS. Purple–pink node bars indicate the 95% HPD. (c) Changes of the effective population size of *S*. Enteritidis ST183 for the NZ isolates. The dark blue line is the mean of the estimated effective population size. The blue-shaded areas are the upper and lower bounds of the 95% HPD interval. The x-axis is the time in years, and the y-axis is population size in log scale. The grey line is the lower 95% HPD boundary for the tree height, while the yellow line is the mean tree height.

Bayesian phylogenetic analyses using BICEPS were applied to the combined ST183 population and the NZ-specific ST183 population. A Bayesian analysis using BICEPS inferred a mean substitution rate of 1.03×10^−7^ substitutions per site per year with a 95% highest posterior density (95% HPD) between 7.0×10^−8^ and 1.3×10^−7^ and Germany as the root population. The mean root height for the combined German, GB and NZ populations was estimated to have a 95% HPD interval between 1856 and 1939. The tMRCA for the GB population was estimated to be between 1926 and 1972 (95% HPD) and for the NZ population was between 1978 and 1998 (95% HPD) (Fig. 3a). The tMRCA for the NZ isolates when analysed on their own was estimated to be in the range 1972–2002 (Fig. 3b) versus 1978–1998 when analysed together with the German and GB isolates. An estimated exponential increase in the NZ ST183 effective population size began in the mid-1990s and flattened to a constant effective population size by the 2010s (Fig. 3c).

## Discussion

### Epidemiology

From January 2020 to December 2023 inclusive, ST183 was the second most notified (44%, 160/367) *S*. Enteritidis ST in NZ. An outbreak of *S*. Enteritidis ST11 (not previously considered an endemic strain) occurred between 2019 and 2023 involving 128 cases [79]. In separate studies in GB and in Denmark, children under the age of four were significantly more likely to be infected with ST183 (or PT11 and PT66) compared with their likelihood of infection with ST11 [5,7]. Our data show that in NZ, children aged 0–4 are at greater risk of *S*. Enteritidis infection irrespective of ST. However, unlike European studies, we found no significant difference between ST183 and non-ST183 (of which ST11 were the majority, 193/207) cases for this age group. People 60 years and older were also at greater risk of *S*. Enteritidis compared to the reference age group, with this pattern being more pronounced among ST183 clinical cases than among non-ST183 clinical cases. One possible explanation for this observation is differential exposure related to time spent in outdoor environments. Previous work has shown that New Zealander adults aged 65 years and older spend an average of 1.09 h outdoors each day, almost double the national summer average over all age groups of 0.55 h per day [80]. This may contribute to increased exposure to environmental sources of ST183 among older adults. However, as specific outdoor activities were not captured in the routinely collected risk factor data, further studies would be required to explore this hypothesis and to better characterize potential exposure pathways.

Genomic sequences of ST183 isolated from a randomly selected diverse range of freshwater sources located in both the North and South Islands of NZ were included in this study and distributed throughout the phylogeny of the NZ isolates. Human cases that had exposure to ‘recreational contact with water’ were significantly lower for ST183 compared to non-ST183. However, there was no significant difference in the subset of these cases exposed to ‘swimming in streams, rivers and sea.’ Isolation of ST183 from these sources does suggest that it is widespread throughout the environment and that recreational water could be a pathway to or from intermediate sources. Unlike non-ST183, no international travel was reported for ST183 cases within the incubation period. This, as well as evidence of ST183 in the environment, confirms that ST183 is endemic to NZ.

### Hedgehogs as reservoir

Previous European studies implicated hedgehogs as a reservoir host species for ST183 [5,7, 13, 15]. Similarly, ST183 was isolated from hedgehogs in NZ in both this study and an earlier study, making it plausible that hedgehogs may serve as a reservoir for ST183 in NZ. Early studies on the distribution of hedgehogs in NZ suggested that they have a strong preference for low-lying grasslands (<800 m) that include both pasture and urban habitats [25,81]. We found that in NZ, ST183 clinical human cases were more likely to live in rural than urban settings, compared with the general population, supporting a rural reservoir. We also found that human cases of ST183 in NZ had proportionally more contact with farm animals than non-ST183 cases. Even though our phylogenetic study did not include farm animals (except for one sheep), transmission between hedgehogs and livestock is plausible because they share the same environment. Studies have shown that hedgehogs prefer a habitat where grass is neither very long nor very short; therefore, their movements are greatly influenced by the grazing pattern of livestock [82]. ST183 isolated from different host species clustered together in the maximum likelihood phylogenetic tree (Fig. 2), supporting interspecies transmissions. This agrees with the findings of Uelze *et al.* [15] and Lawson *et al.* [7]. However, it is not possible to infer direct transmission from whole genome sequencing(WGS) data alone [83]. Because hedgehogs can act as a reservoir for ST183, they might contribute toward its persistence and spread in the environment or livestock settings in NZ. In addition, hedgehogs will scavenge on any carrion and carrion feeding insects such as maggots, potentially contributing to the spread of infection [84].

Consideration should be given to the difference in the status of hedgehogs in NZ versus Europe and the impact of sampling strategies and study outcomes. Although a synanthropic species, unlike in Europe, sick hedgehogs are seldom taken into human care in NZ as they are considered pests and detrimental to NZ native wildlife. During a previous study in NZ (1995), *S*. Enteritidis PT9a was isolated from eight of 202 hedgehogs tested [23]. However, no other *S*. Enteritidis PT was isolated. In addition, this previous study showed regional differences in infection with a higher prevalence for *S*. Enteritidis in hedgehogs from Wairarapa than in the other regions tested (North Canterbury and Otago) [23]. Our study isolated ST183 from 3 out of 45 hedgehog carcasses sampled, and no other *S*. Enteritidis or *Salmonella* serovar was isolated. These were collected from November 2023 to February 2024 inclusive, one from the Canterbury region while two were from Waikato. Even though the small scale of our study is a clear limitation, it indicates an ongoing presence of ST183 in the NZ hedgehog population. Further studies are needed to determine the full role of hedgehogs as a reservoir for ST183.

### AMR, plasmids and pathogenicity islands

Our study also found that all six NZ clinical human isolates selected were susceptible to the antimicrobial agents tested phenotypically including the aminoglycoside gentamicin. This agrees with earlier studies that found no phenotypic evidence of AMR for the panel that was tested, from *S*. Enteritidis PT11 or ST183 in hedgehogs [7,15, 85]. However, Humphries argued that aminoglycosides may appear active *in vitro* against *Salmonella* but are not clinically effective [86]. Both *mdsA* and *mdsB* were detected in most isolates tested during our study. However, without the presence of *mdsC* or *tolC*, the multidrug efflux pump is incomplete, which is in line with phenotypic susceptibility data observed for the ST183 isolates by Uelze *et al.* [15].

*S. enterica* can persist for extended periods in non-host environments, including soil, water and agricultural settings [87]. In *Salmonella*, the gold-responsive transcriptional regulator GolS detects intracellular Au(I) ions and activates a hierarchically organized regulon that includes golT, encoding a P-type ATPase, and controls the expression of the RND efflux pump GesABC that enables the active export of toxic gold ions, allowing *Salmonella* to tolerate and persist in gold-contaminated environments [88,89]. The *gol* locus is conserved within the genus *Salmonella*, being present in both *S. bongori* and *S. enterica*, but is absent from other enteric bacteria including *Escherichia coli*, *Shigella* and *Yersinia* [88]. The specificity of GolS for gold, rather than copper or silver, suggests that the gol system represents a specialized environmental adaptation, facilitating survival in metal-rich soil or sedimentary niches rather than serving a primary role in host-associated copper homeostasis [90]. Our study is consistent with previous studies by Cao *et al.* who detected IncFII(S) in four out of five ST183 genomes tested and Uelze *et al.* who predicted a plasmid size of 87,300 bp [IncFII(S)_1] in all wildlife ST183 [15,85]. Presence of plasmids belonging to the IncF plasmid family (often in multi-replicon variants) is commonly described in different *Salmonella* serovars and often associated with AMR or virulence factors. Moreover, IncF is one of the main incompatibility groups associated with well-described virulence plasmids in *Salmonella,* such as pSPV in *S*. Gallinarum/Pullorum and pSTV in *S*. Typhimurium lineages [91]. Since virulence plasmids in *S*. Enteritidis are known to lack an intact transfer region, the detection of additional ColE-like plasmids can compensate for this non-transferability through their capability to mobilize other plasmids [91].

### WGS SNP phylogenetic analysis of bacterial populations in NZ, Germany and GB

The maximum likelihood phylogenetic tree in combination with hierarchical clustering showed separate but multiple segregated clusters for each country of origin. The monophyletic nature of the NZ cluster is suggestive of a single introduction into NZ just prior to the first detection. However, multiple introductions into NZ earlier, followed by bottleneck event(s), cannot be ruled out. Our study supports and extends that of Uelze *et al.* in that isolates cluster strongly according to country of origin and that isolation year does not affect clustering results [15]. Consistent with other studies, we found that different host species, including hedgehogs, clustered together, suggesting interspecies transmission events [7]. All ST183 isolates related to a regional outbreak in 2021 clustered together (within 0 or one core SNP difference) with an isolate from the implicated food source: alfalfa sprouts [9,11]. Alfalfa sprout has often been implicated in *Salmonella* outbreaks worldwide due to the contamination of seeds [92,93]. Howard *et al.* showed that *Salmonella* are often found epiphytically at very low levels on sprout seeds. However, germinating alfalfa seeds support the multiplication of *S. enterica,* independent of serovar, prior to the emergence of the root radicle at 72 h to clinically significant populations [94].

### Bayesian phylogenetic analysis

Bayesian temporal analysis was used to address the question of whether the ancestral lineage of the current circulating ST183 strains in NZ was associated with the introduction of hedgehogs from GB in the late 19th century.

A historic account of *S*. Enteritidis (ST and/or PT unknown) in NZ suggests that it was first isolated between 1967 and 1972 from two dogs in the Waikato region [95]. Phage typing of animal isolates of *S*. Enteritidis in NZ started in 1986, and most were PT9a. Initially, only sporadic isolates of *S*. Enteritidis were recovered: one from cattle in 1988 and one each from sheep and sewage sludge in 1990 [95]. During the period 1988–1990, PHF Science identified serovars of *S. enterica* subsp. *enterica* from 610 poultry sources and 943 other domestic animals. It has been reported that from 1990, isolates of *S*. Enteritidis became more frequent [95]. While annual notifications of human cases of *S*. Enteritidis remained in the range of 10–20 from 1985 to 1989, they had increased to about 150 by 1994 [95]. International travel data is not available for these historic cases. In addition, Gorton *et al.* isolated *S*. Enteritidis PT9a from hedgehogs in NZ in 1995 [23].

Our study estimates that the current circulating lineage of ST183 in NZ has a tMRCA between 1972 and 2002 (95% HPD). This is consistent with the first-time ST183-associated PTs being isolated from human clinical samples in NZ [95]. Despite uncertainty of the trajectory, our study estimated an exponential population size increase for ST183 between the 1990s to the 2010s, once again agreeing with historical accounts [95]. The reason for this increase is unclear. Our study estimated a mean substitution rate of 1.03×10^−7^ substitutions per site per year for the combined ST183 populations of Germany, GB and NZ. This substitution rate is lower than previously estimated for *S*. Enteritidis [76,96]. However, Luo *et al.* reported that different strains of *S*. Enteritidis, even of the same ST, may have significantly different substitution rates [97]. For *S*. Typhimurium PT160, Bloomfield *et al.* estimated 3.3–4.3×10^–7^ [98], which is higher than described for any of the *S*. Enteritidis substitution rates.

One can propose a delayed spread of ST183 after the first recorded successful introduction in 1881 of hedgehogs, followed by a period when ST183 was stably maintained in the environment and other new reservoirs, such as farmed animals. Since the hedgehog populations finally began to increase in the 1930s, a subsequent spread of ST183 and thus detectability based on prevalence is expected. However, deviations between this hypothesis and the available epidemiological data and the Bayesian temporal analysis do not support this assumption. While an extended period for the pathogen to settle into the NZ environment cannot be excluded, the introduction of hedgehogs to NZ (1880s sourced from GB) predates even the tMRCA for the current circulating strain of ST183 in GB (1926–1972). Hence, a later introduction of ST183 to NZ, as shown by our study, seems more plausible.

Even though previous European studies implicated hedgehogs as a potential reservoir host species for ST183, the epidemiological evidence in NZ identifies contact with farm animals as a risk factor. Therefore, it is plausible that farm animals are vehicles of introduction, reservoirs or bridge hosts of ST183. Between 2010 and 2019 inclusive, PHF Science isolated ST183 (PT9a and PT11) from 1 chicken, 42 cows, 9 dogs, 2 goats, 5 horses, 8 cats, 5 sheep samples and 4 from the environment (unspecified) [22]. A large number of live sheep (*n*=6,712) and cattle (*n*=3,909) were imported from the UK between 1868 and 1979 [99]. In the early 1970s, there was a sharp increase in the import of cattle stock mainly from Australia and GB, partly driven by short-lived favourable market conditions, a drop in wool prices in the late 1960s and the NZ government’s incentives for intensification in farming [99,101]. However, by 1977, the size of NZ cattle herds and meat production fell due to various reasons including market pressure [102,103]. The MRCA for ST183 in NZ coincides with the sharp increase in the importation of livestock in the early 1970s. Previous studies have shown an association between this period of livestock importation into NZ and the introduction of zoonotic pathogens such as *E. coli* O26 and O157 [104,105]. Our study shows that the current circulating NZ strain of ST183 is more closely related to the GB population than the German population (Fig. 3). However, inclusion of ST183 sequence data from countries not included in this study may provide inference of alternative transmission pathways.

### Limitation and future direction

While complete phenotypic antimicrobial susceptibility is reassuring and consistent with earlier studies, a small sample size increases the risk that rare or emerging resistance phenotypes were not detected. These isolates may not capture the full genetic or ecological diversity of ST183, particularly across different hosts. Sampling of hedgehogs was limited to 45 animals from a small number of regions in NZ, yielding only 3 ST183 isolates; therefore, inferences from such data are rather restrictive. In addition, variability in the timing of trap checks, storage conditions and transport of carcasses to central facilities meant that these factors could not be standardized. As a result, prevalence estimation was not a primary objective of this study and is recognized as a limitation. However, this study provides sound pilot data to inform future larger-scale investigations and wildlife surveillance strategies. Potential occurrence of direct or indirect contact between clinical human cases and hedgehogs requires further investigation to understand the risk associated. For example, ‘contact with wildlife’ should be considered for inclusion as a routine question on future public health questionnaires. Epidemiological evidence identified ‘contact with farm animals’ as a significant risk factor, although very few ST183 isolates derived from livestock were included for phylodynamic analysis. Temporal phylogenetic analysis of ST183, in combination with a better understanding of the prevalence of ST183 in NZ livestock, will potentially provide more information on how past events shaped the evolutionary trajectory of this microbial pathogen.

## Conclusion

This study found no association between the current circulating strain of ST183 and the introduction of hedgehogs to NZ. Analyses instead show that the current circulating strain of ST183 was first introduced to NZ around a century later than hedgehogs. Our study showed that ST183 is present in hedgehogs in NZ. Although the small sample size did not provide definitive evidence that hedgehogs act as reservoir hosts for ST183 in NZ, their role as a reservoir has been suggested in other studies in mainland Europe and GB. Our data support interspecies transmission and suggest that ST183 is widespread throughout the NZ environment. Based on current knowledge, we recommend an enhanced One Health approach to *Salmonella* surveillance to inform infection sources and pathways in NZ and future public health strategies. We propose that hedgehogs in NZ should be considered a temporal sentinel of the prevalence of ST183 in the environment.

## Supplementary material

10.1099/mgen.0.001677 Uncited Supplementary Material 1.

## Abbreviations

AMR: antimicrobial resistance
95% CI: 95% confidence interval
ERL: Enteric Reference Laboratory
EUCAST: European Committee on Antimicrobial Susceptibility Testing
GB: Great Britain
HE: Hektoen enteric
HKY: Hasegawa–Kishino–Yano
95% HPD: 95% highest posterior density
iTOL: Interactive Tree of Life
MCMC: Markov chain Monte Carlo
MLST: multilocus sequence typing
NZ: New Zealand
PHF Science: New Zealand Institute for Public Health and Forensic Science
PT: phage type
RKI: Robert Koch Institute
RND: resistance–nodulation–division
RR: relative rates
ST: sequence type
tMRCA: time to the Most Recent Common Ancestor
TSA: tryptic soy agar
VFDB: Virulence Factors Database
XLD: xylose lysine deoxycholate
