# Supplementary material for: Phylodynamic analysis of Salmonella Enteritidis ST183 in Aotearoa New Zealand finds no evidence for introduction via European hedgehogs (Erinaceus europaeus)
Source: Microb Genom. 2026 Mar 18;12(3):001677. doi: 10.1099/mgen.0.001677 (PMC12999276; doi:10.1099/mgen.0.001677)
Supplement: Uncited Supplementary Material 1. [file mgen-12-01677-s001.pdf]

Phylodynamic analysis of *Salmonella* Enteritidis ST183 in Aotearoa New Zealand finds no evidence for introduction via European hedgehogs (*Erinaceus europaeus*).

Supplementary material

Table S1. Genomic sequences of ST183 isolates included in this study, *n* = 448

SRA accession	Collection date	2021 NZ outbreak	Host	Country	coreSNP analysis	Temporal phylogenetic reconstruction
SRR1969448	3/11/2013	-	hedgehog	GB	✓	✓
SRR1966855	24/04/2014	-	hedgehog	GB	✓	✓
SRR1966231	30/04/2014	-	hedgehog	GB	✓	✓
SRR1958139	10/05/2014	-	hedgehog	GB	✓	✓
SRR1970181	23/08/2014	-	hedgehog	GB	✓	✓
SRR1966154	1/10/2014	-	hedgehog	GB	✓	✓
SRR5757189	4/10/2014	-	hedgehog	GB	✓	✓
SRR5757187	23/12/2014	-	hedgehog	GB	✓	✓
SRR5814207	28/07/2015	-	hedgehog	GB	✓	✓
SRR5193311	20/08/2015	-	hedgehog	GB	✓	✓
SRR5757207	28/08/2015	-	hedgehog	GB	✓	✓
SRR5757200	31/08/2015	-	hedgehog	GB	✓	✓
SRR5220914	8/09/2015	-	hedgehog	GB	✓	✓
SRR5757176	8/09/2015	-	hedgehog	GB	✓	✓
SRR5220891	18/09/2015	-	hedgehog	GB	✓	✓
SRR5757192	25/09/2015	-	hedgehog	GB	✓	
SRR5216613	25/10/2015	-	hedgehog	GB	✓	✓
SRR5757213	27/10/2015	-	hedgehog	GB	✓	✓
SRR5220842	1/11/2015	-	hedgehog	GB	✓	✓
SRR5757178	3/11/2015	-	hedgehog	GB	✓	✓
SRR5193500	13/12/2015	-	hedgehog	GB	✓	✓
SRR3322670	Unknown	-	hedgehog	GB	✓	
SRR5193740	Unknown	-	hedgehog	GB	✓	
SRR5814202	22/07/2012	-	human	GB	✓	✓
SRR5814201	31/07/2012	-	human	GB	✓	
SRR6326695	16/08/2012	-	human	GB	✓	
SRR1645784	21/08/2012	-	human	GB	✓	✓
SRR5814203	23/08/2012	-	human	GB	✓	
SRR5814204	6/09/2012	-	human	GB	✓	
SRR5814205	27/09/2012	-	human	GB	✓	✓
SRR5814208	11/10/2012	-	human	GB	✓	
SRR5814206	25/10/2012	-	human	GB	✓	
SRR1966922	16/04/2014	-	human	GB	✓	
SRR3049808	17/04/2014	-	human	GB	✓	
SRR1969624	7/05/2014	-	human	GB	✓	
SRR1966425	21/05/2014	-	human	GB	✓	
SRR3048712	22/05/2014	-	human	GB	✓	
SRR3049205	30/05/2014	-	human	GB	✓	
SRR3048839	1/07/2014	-	human	GB	✓	

SRA accession	Collection date	2021 NZ outbreak	Host	Country	coreSNP analysis	Temporal phylogenetic reconstruction
SRR1969918	2/07/2014	-	human	GB	✓	
SRR3048925	11/07/2014	-	human	GB	✓	
SRR3049774	15/07/2014	-	human	GB	✓	
SRR3049837	16/07/2014	-	human	GB	✓	
SRR1968131	24/07/2014	-	human	GB	✓	✓
SRR1966925	31/07/2014	-	human	GB	✓	
SRR1968103	1/08/2014	-	human	GB	✓	
SRR1965640	12/08/2014	-	human	GB	✓	
SRR1969040	12/08/2014	-	human	GB	✓	
SRR1958647	15/08/2014	-	human	GB	✓	✓
SRR1965437	15/08/2014	-	human	GB	✓	
SRR1969670	16/08/2014	-	human	GB	✓	
SRR1965923	17/08/2014	-	human	GB	✓	
SRR3049108	17/08/2014	-	human	GB	✓	
SRR3049114	17/08/2014	-	human	GB	✓	✓
SRR3049525	20/08/2014	-	human	GB	✓	
SRR1957878	25/08/2014	-	human	GB	✓	
SRR3049418	29/08/2014	-	human	GB	✓	✓
SRR1958210	1/09/2014	-	human	GB	✓	
SRR1957742	2/09/2014	-	human	GB	✓	
SRR1957804	8/09/2014	-	human	GB	✓	
SRR1966056	15/09/2014	-	human	GB	✓	✓
SRR1958392	16/09/2014	-	human	GB	✓	
SRR1958410	16/09/2014	-	human	GB	✓	
SRR1957880	18/09/2014	-	human	GB	✓	
SRR1958383	22/09/2014	-	human	GB	✓	✓
SRR1958484	22/09/2014	-	human	GB	✓	
SRR3049710	22/09/2014	-	human	GB	✓	
SRR1958370	30/09/2014	-	human	GB	✓	
SRR1967654	30/09/2014	-	human	GB	✓	
SRR1968477	2/10/2014	-	human	GB	✓	
SRR1969780	2/10/2014	-	human	GB	✓	
SRR3049772	2/10/2014	-	human	GB	✓	✓
SRR1965630	3/10/2014	-	human	GB	✓	
SRR1958610	4/10/2014	-	human	GB	✓	
SRR1966114	6/10/2014	-	human	GB	✓	
SRR1957886	7/10/2014	-	human	GB	✓	✓
SRR1958091	7/10/2014	-	human	GB	✓	
SRR1969169	7/10/2014	-	human	GB	✓	✓
SRR1967015	13/10/2014	-	human	GB	✓	
SRR1969903	13/10/2014	-	human	GB	✓	
SRR1959307	20/10/2014	-	human	GB	✓	
SRR1966860	21/10/2014	-	human	GB	✓	
SRR1965918	4/11/2014	-	human	GB	✓	
SRR1968500	5/11/2014	-	human	GB	✓	
SRR1963148	6/11/2014	-	human	GB	✓	
SRR1969909	10/11/2014	-	human	GB	✓	

SRA accession	Collection date	2021 NZ outbreak	Host	Country	coreSNP analysis	Temporal phylogenetic reconstruction
SRR1963317	14/11/2014	-	human	GB	✓	
SRR1963453	20/11/2014	-	human	GB	✓	
SRR1961507	8/12/2014	-	human	GB	✓	
SRR5757198	19/04/2015	-	human	GB	✓	
SRR5216505	24/04/2015	-	human	GB	✓	
SRR5220600	6/05/2015	-	human	GB	✓	
SRR5813587	18/05/2015	-	human	GB	✓	
SRR5193320	26/05/2015	-	human	GB	✓	
SRR5813588	8/06/2015	-	human	GB	✓	
SRR5814210	15/06/2015	-	human	GB	✓	
SRR3585361	29/06/2015	-	human	GB	✓	
SRR3322692	30/06/2015	-	human	GB	✓	
SRR5194101	1/07/2015	-	human	GB	✓	
SRR3321885	4/07/2015	-	human	GB	✓	
SRR5757205	7/07/2015	-	human	GB	✓	✓
SRR3284749	9/07/2015	-	human	GB	✓	
SRR5216466	9/07/2015	-	human	GB	✓	
SRR5220195	9/07/2015	-	human	GB	✓	
SRR5757182	9/07/2015	-	human	GB	✓	
SRR5757211	9/07/2015	-	human	GB	✓	
SRR3322002	13/07/2015	-	human	GB	✓	
SRR3322988	14/07/2015	-	human	GB	✓	
SRR3285341	21/07/2015	-	human	GB	✓	
SRR5757210	23/07/2015	-	human	GB	✓	
SRR5216508	30/07/2015	-	human	GB	✓	
SRR5220609	31/07/2015	-	human	GB	✓	
SRR5757199	5/08/2015	-	human	GB	✓	
SRR5757186	6/08/2015	-	human	GB	✓	
SRR5216517	11/08/2015	-	human	GB	✓	
SRR3322958	19/08/2015	-	human	GB	✓	
SRR5757190	20/08/2015	-	human	GB	✓	
SRR3585350	21/08/2015	-	human	GB	✓	
SRR5757215	27/08/2015	-	human	GB	✓	
SRR3322078	28/08/2015	-	human	GB	✓	
SRR5193739	28/08/2015	-	human	GB	✓	
SRR5757185	28/08/2015	-	human	GB	✓	
SRR5757204	28/08/2015	-	human	GB	✓	
SRR5216432	3/09/2015	-	human	GB	✓	✓
SRR5216610	4/09/2015	-	human	GB	✓	✓
SRR5216634	6/09/2015	-	human	GB	✓	
SRR5215853	14/09/2015	-	human	GB	✓	
SRR5216325	21/09/2015	-	human	GB	✓	
SRR3285375	22/09/2015	-	human	GB	✓	✓
SRR5220608	27/09/2015	-	human	GB	✓	
SRR5193937	28/09/2015	-	human	GB	✓	
SRR5757197	28/09/2015	-	human	GB	✓	✓
SRR5757201	28/09/2015	-	human	GB	✓	

SRA accession	Collection date	2021 NZ outbreak	Host	Country	coreSNP analysis	Temporal phylogenetic reconstruction
SRR3285081	30/09/2015	-	human	GB	✓	
SRR5757212	30/09/2015	-	human	GB	✓	✓
SRR5193846	2/10/2015	-	human	GB	✓	
SRR5757209	8/10/2015	-	human	GB	✓	
SRR5757194	16/10/2015	-	human	GB	✓	
SRR5220251	29/10/2015	-	human	GB	✓	✓
SRR5216207	5/11/2015	-	human	GB	✓	
SRR3284676	9/11/2015	-	human	GB	✓	
SRR5193234	10/11/2015	-	human	GB	✓	
SRR5757179	21/11/2015	-	human	GB	✓	
SRR5193757	23/11/2015	-	human	GB	✓	
SRR5215712	30/11/2015	-	human	GB	✓	
SRR5215850	2/12/2015	-	human	GB	✓	
SRR5757196	17/03/2016	-	human	GB	✓	✓
SRR35834416	31/07/2018	-	cat	Germany	✓	✓
SRR35834354	8/08/2019	-	cat	Germany	✓	
SRR35834352	18/09/2019	-	cat	Germany	✓	
SRR35834347	24/08/2020	-	cat	Germany	✓	✓
SRR35834409	22/09/2020	-	cat	Germany	✓	
ERR3581164	24/09/2018	-	chicken	Germany	✓	
SRR35834356	12/03/2019	-	chicken	Germany	✓	
SRR35834345	8/09/2020	-	chicken	Germany	✓	
SRR35834410	16/09/2020	-	chicken	Germany	✓	
SRR35834340	28/10/2020	-	chicken	Germany	✓	
SRR35834387	13/12/2021	-	chicken	Germany	✓	
SRR35834415	10/09/2018	-	dog	Germany	✓	
ERR3580943	20/12/2018	-	dog	Germany	✓	✓
SRR35834348	25/06/2020	-	dog	Germany	✓	
SRR35834403	27/02/2021	-	dog	Germany	✓	
SRR35834373	26/10/2022	-	dog	Germany	✓	✓
SRR35834351	30/11/2016	-	fox	Germany	✓	
SRR35834365	17/08/2018	-	fox	Germany	✓	
SRR31567508	12/08/2021	-	fox	Germany	✓	
SRR35834384	2/03/2022	-	fox	Germany	✓	
SRR35834374	24/10/2022	-	fox	Germany	✓	
ERR3581020	1/01/2018	-	goat	Germany	✓	
SRR14998797	27/11/2008	-	grey heron	Germany	✓	
ERR3581016	27/12/2017	-	hedgehog	Germany	✓	✓
ERR3580905	12/01/2018	-	hedgehog	Germany	✓	
ERR3580819	18/06/2018	-	hedgehog	Germany	✓	
ERR3581019	25/07/2018	-	hedgehog	Germany	✓	
ERR3581818	23/08/2018	-	hedgehog	Germany	✓	
ERR3581833	19/09/2018	-	hedgehog	Germany	✓	
SRR35834362	27/09/2018	-	hedgehog	Germany	✓	
ERR3581434	28/09/2018	-	hedgehog	Germany	✓	✓
ERR3581768	28/09/2018	-	hedgehog	Germany	✓	
ERR3580804	15/10/2018	-	hedgehog	Germany	✓	

SRA accession	Collection date	2021 NZ outbreak	Host	Country	coreSNP analysis	Temporal phylogenetic reconstruction
SRR35834359	17/10/2018	-	hedgehog	Germany	✓	✓
SRR35834360	17/10/2018	-	hedgehog	Germany	✓	
SRR35834366	18/10/2018	-	hedgehog	Germany	✓	
ERR3580952	29/10/2018	-	hedgehog	Germany	✓	
SRR35834358	19/11/2018	-	hedgehog	Germany	✓	
SRR14998842	15/07/2019	-	hedgehog	Germany	✓	
SRR14998829	10/09/2019	-	hedgehog	Germany	✓	✓
SRR35834411	20/09/2019	-	hedgehog	Germany	✓	
SRR35834353	1/10/2019	-	hedgehog	Germany	✓	
SRR14998837	6/10/2019	-	hedgehog	Germany	✓	
SRR14998832	8/11/2019	-	hedgehog	Germany	✓	
SRR14998833	19/11/2019	-	hedgehog	Germany	✓	
SRR35834400	26/11/2019	-	hedgehog	Germany	✓	
SRR35834349	31/01/2020	-	hedgehog	Germany	✓	
SRR14998993	18/02/2020	-	hedgehog	Germany	✓	
SRR35834412	10/09/2020	-	hedgehog	Germany	✓	
SRR14998943	28/09/2020	-	hedgehog	Germany	✓	✓
SRR14998953	28/09/2020	-	hedgehog	Germany	✓	
SRR14998951	4/10/2020	-	hedgehog	Germany	✓	
SRR14998942	9/10/2020	-	hedgehog	Germany	✓	
SRR14998941	13/10/2020	-	hedgehog	Germany	✓	✓
SRR35834408	21/10/2020	-	hedgehog	Germany	✓	
SRR14998939	22/10/2020	-	hedgehog	Germany	✓	
SRR35834413	26/10/2020	-	hedgehog	Germany	✓	
SRR35834414	27/11/2020	-	hedgehog	Germany	✓	
SRR35834378	11/03/2021	-	hedgehog	Germany	✓	
SRR35834398	9/07/2021	-	hedgehog	Germany	✓	
SRR35834401	24/09/2021	-	hedgehog	Germany	✓	✓
SRR35834397	16/10/2021	-	hedgehog	Germany	✓	
SRR35834390	3/11/2021	-	hedgehog	Germany	✓	
SRR35834388	10/11/2021	-	hedgehog	Germany	✓	
SRR35834395	11/11/2021	-	hedgehog	Germany	✓	
SRR35834393	29/11/2021	-	hedgehog	Germany	✓	
SRR35834391	13/12/2021	-	hedgehog	Germany	✓	
SRR35834385	31/01/2022	-	hedgehog	Germany	✓	
SRR35834383	9/05/2022	-	hedgehog	Germany	✓	✓
SRR35834377	7/08/2022	-	hedgehog	Germany	✓	
SRR35834367	14/09/2022	-	hedgehog	Germany	✓	✓
SRR35834375	17/10/2022	-	hedgehog	Germany	✓	
ERR15726043	14/09/2015	-	human	Germany	✓	✓
ERR15726044	7/12/2015	-	human	Germany	✓	
ERR15726045	19/05/2016	-	human	Germany	✓	✓
ERR15726046	22/06/2016	-	human	Germany	✓	
ERR15726047	27/12/2018	-	human	Germany	✓	✓
ERR15726048	28/10/2019	-	human	Germany	✓	✓
ERR15726049	30/10/2019	-	human	Germany	✓	
ERR15726050	4/11/2019	-	human	Germany	✓	✓

SRA accession	Collection date	2021 NZ outbreak	Host	Country	coreSNP analysis	Temporal phylogenetic reconstruction
ERR15726051	13/11/2019	-	human	Germany	✓	
ERR15726052	12/06/2020	-	human	Germany	✓	✓
ERR15726053	15/06/2020	-	human	Germany	✓	
ERR15726054	1/07/2020	-	human	Germany	✓	
ERR15726055	15/07/2020	-	human	Germany	✓	✓
ERR15726056	3/08/2020	-	human	Germany	✓	
ERR15726057	3/08/2020	-	human	Germany	✓	
ERR15726058	6/08/2020	-	human	Germany	✓	
ERR15726059	12/08/2020	-	human	Germany	✓	✓
ERR15726060	13/08/2020	-	human	Germany	✓	
ERR15726061	20/08/2020	-	human	Germany	✓	✓
ERR15726062	21/08/2020	-	human	Germany	✓	
ERR15726063	24/08/2020	-	human	Germany	✓	
ERR15726064	9/09/2020	-	human	Germany	✓	
ERR15726065	11/09/2020	-	human	Germany	✓	
ERR15726066	30/09/2020	-	human	Germany	✓	✓
ERR15726067	7/10/2020	-	human	Germany	✓	✓
ERR15726068	21/10/2020	-	human	Germany	✓	
ERR15726069	22/10/2020	-	human	Germany	✓	
ERR15726070	30/10/2020	-	human	Germany	✓	✓
ERR15726071	9/11/2020	-	human	Germany	✓	
ERR15726072	20/11/2020	-	human	Germany	✓	
ERR15726073	22/06/2021	-	human	Germany	✓	
ERR15726074	28/06/2021	-	human	Germany	✓	✓
ERR15726075	29/06/2021	-	human	Germany	✓	
ERR15726076	7/07/2021	-	human	Germany	✓	
ERR15726077	12/07/2021	-	human	Germany	✓	
ERR15726078	20/07/2021	-	human	Germany	✓	
ERR15726079	28/07/2021	-	human	Germany	✓	✓
ERR15726080	6/09/2021	-	human	Germany	✓	
ERR15726081	17/09/2021	-	human	Germany	✓	
ERR15726082	27/09/2021	-	human	Germany	✓	
ERR15726083	27/09/2021	-	human	Germany	✓	
ERR15726084	30/09/2021	-	human	Germany	✓	✓
ERR15726085	6/10/2021	-	human	Germany	✓	
ERR15726086	20/10/2021	-	human	Germany	✓	
ERR15726087	11/05/2022	-	human	Germany	✓	
ERR15726088	20/07/2022	-	human	Germany	✓	✓
ERR15726089	29/07/2022	-	human	Germany	✓	
ERR15726090	15/08/2022	-	human	Germany	✓	
ERR15726091	23/08/2022	-	human	Germany	✓	✓
ERR15726092	1/09/2022	-	human	Germany	✓	
ERR15726093	12/09/2022	-	human	Germany	✓	✓
ERR15726094	25/10/2022	-	human	Germany	✓	
ERR15726095	11/11/2022	-	human	Germany	✓	
ERR15726096	1/12/2022	-	human	Germany	✓	✓
ERR15726097	13/01/2023	-	human	Germany	✓	

SRA accession	Collection date	2021 NZ outbreak	Host	Country	coreSNP analysis	Temporal phylogenetic reconstruction
ERR15726098	7/03/2023	-	human	Germany	✓	✓
SRR35834346	9/09/2020	-	livestock	Germany	✓	
SRR35834379	7/07/2022	-	mammals, faeces	Germany	✓	✓
SRR35834350	12/03/2020	-	meat product	Germany	✓	
SRR35834404	17/12/2020	-	mixed herbs	Germany	✓	
SRR35834405	18/12/2020	-	mixed herbs	Germany	✓	
SRR35834381	10/06/2022	-	mouse	Germany	✓	
SRR35834368	14/09/2018	-	pig	Germany	✓	
SRR35834396	16/10/2021	-	raccoon	Germany	✓	
SRR14998775	10/12/2008	-	red fox	Germany	✓	
SRR14998892	24/08/2016	-	red fox	Germany	✓	✓
SRR35834399	16/09/2021	-	reptile	Germany	✓	
SRR35834380	10/06/2022	-	reptile	Germany	✓	
SRR14998920	1/10/2007	-	roe deer	Germany	✓	✓
SRR14998971	3/02/2009	-	roe deer	Germany	✓	✓
SRR14998904	4/05/2015	-	rook	Germany	✓	✓
SRR35834382	9/05/2022	-	sheep	Germany	✓	
ERR3580790	31/08/2018	-	turkey	Germany	✓	
SRR14998819	29/09/2008	-	wild boar	Germany	✓	✓
SRR35834386	12/01/2022	-	wild boar	Germany	✓	
SRR35834344	14/07/2017	-	wildlife	Germany	✓	✓
SRR35834364	20/07/2017	-	wildlife	Germany	✓	
SRR35834406	28/12/2020	-	wildlife	Germany	✓	
SRR35834392	27/11/2021	-	zoo animals	Germany	✓	
SRR35834376	11/10/2022	-	zoo animals	Germany	✓	
SRR35901116	17/04/2013	-	cat	NZ	✓	
SRR35901126	6/04/2021	-	cat	NZ	✓	✓
SRR35901114	30/12/2021	-	cat	NZ	✓	✓
SRR35901113	20/12/2021	-	dog	NZ	✓	✓
SRR35901127	9/02/2021	-	Food (sprouts)	NZ	✓	
SRR35901108	3/02/2020	-	freshwater	NZ	✓	✓
SRR35901109	3/02/2020	-	freshwater	NZ	✓	✓
SRR35901125	16/03/2020	-	freshwater	NZ	✓	✓
SRR35901123	15/03/2021	-	freshwater	NZ	✓	✓
SRR35901124	22/03/2021	-	freshwater	NZ	✓	
SRR35901122	29/03/2021	-	freshwater	NZ	✓	
SRR35901121	12/04/2021	-	freshwater	NZ	✓	✓
SRR35901120	17/05/2021	-	freshwater	NZ	✓	✓
SRR35901119	28/01/2002	-	hedgehog	NZ	✓	✓
SRR35901118	31/01/2006	-	hedgehog	NZ	✓	✓
SRR35901117	2/03/2006	-	hedgehog	NZ	✓	✓
SRR35901111	24/12/2023	-	hedgehog	NZ	✓	✓
SRR35901112	1/12/2023*	-	hedgehog	NZ	✓	✓
SRR35901110	30/01/2024*	-	hedgehog	NZ	✓	✓
SRR35900374	2/08/2012	-	human	NZ	✓	
SRR35900449	20/06/2013	-	human	NZ	✓	✓
SRR35900448	14/12/2013	-	human	NZ	✓	✓

SRA accession	Collection date	2021 NZ outbreak	Host	Country	coreSNP analysis	Temporal phylogenetic reconstruction
SRR35900437	29/04/2014	-	human	NZ	✓	
SRR35900426	28/11/2014	-	human	NZ	✓	
SRR35900391	21/04/2015	-	human	NZ	✓	✓
SRR35900380	24/12/2015	-	human	NZ	✓	✓
SRR35900406	24/02/2016	-	human	NZ	✓	
SRR35900417	28/02/2016	-	human	NZ	✓	✓
SRR35900373	7/03/2016	-	human	NZ	✓	
SRR35900372	15/03/2016	-	human	NZ	✓	
SRR35900447	16/05/2016	-	human	NZ	✓	✓
SRR35900446	18/11/2019	-	human	NZ	✓	✓
SRR35900444	31/01/2020	-	human	NZ	✓	
SRR35900445	31/01/2020	-	human	NZ	✓	✓
SRR35900443	6/02/2020	-	human	NZ	✓	
SRR35900441	13/02/2020	-	human	NZ	✓	
SRR35900442	13/02/2020	-	human	NZ	✓	
SRR35900438	2/03/2020	-	human	NZ	✓	
SRR35900440	6/03/2020	-	human	NZ	✓	✓
SRR35900439	30/03/2020	-	human	NZ	✓	
SRR35900436	10/04/2020	-	human	NZ	✓	
SRR35900435	29/04/2020	-	human	NZ	✓	✓
SRR35900434	7/05/2020	-	human	NZ	✓	
SRR35900433	18/05/2020	-	human	NZ	✓	
SRR35900432	4/06/2020	-	human	NZ	✓	✓
SRR35900431	19/08/2020	-	human	NZ	✓	
SRR35900430	21/09/2020	-	human	NZ	✓	
SRR35900429	28/09/2020	-	human	NZ	✓	✓
SRR35900428	30/09/2020	-	human	NZ	✓	
SRR35900427	27/10/2020	-	human	NZ	✓	
SRR35900401	30/11/2020	-	human	NZ	✓	
SRR35900400	1/12/2020	-	human	NZ	✓	✓
SRR14716964	9/01/2021	-	human	NZ	✓	✓
SRR14716963	11/01/2021	-	human	NZ	✓	
SRR14716909	26/01/2021	-	human	NZ	✓	
SRR14716978	26/01/2021	-	human	NZ	✓	
SRR14716902	28/01/2021	Yes	human	NZ	✓	
SRR14716904	28/01/2021	-	human	NZ	✓	
SRR14716906	28/01/2021	Yes	human	NZ	✓	
SRR14716982	28/01/2021	Yes	human	NZ	✓	
SRR14716913	29/01/2021	Yes	human	NZ	✓	
SRR14716918	29/01/2021	Yes	human	NZ	✓	
SRR14716924	29/01/2021	Yes	human	NZ	✓	
SRR14716921	30/01/2021	Yes	human	NZ	✓	
SRR14716916	1/02/2021	Yes	human	NZ	✓	
SRR14716917	1/02/2021	Yes	human	NZ	✓	
SRR14716922	1/02/2021	Yes	human	NZ	✓	
SRR14716923	1/02/2021	Yes	human	NZ	✓	
SRR14716928	1/02/2021	Yes	human	NZ	✓	

SRA accession	Collection date	2021 NZ outbreak	Host	Country	coreSNP analysis	Temporal phylogenetic reconstruction
SRR14716933	1/02/2021	-	human	NZ	✓	
SRR14716915	2/02/2021	Yes	human	NZ	✓	
SRR14716926	2/02/2021	Yes	human	NZ	✓	
SRR14716927	4/02/2021	Yes	human	NZ	✓	
SRR14716930	4/02/2021	Yes	human	NZ	✓	
SRR14716934	4/02/2021	Yes	human	NZ	✓	
SRR14716935	4/02/2021	Yes	human	NZ	✓	
SRR14716980	4/02/2021	Yes	human	NZ	✓	
SRR14716938	9/02/2021	Yes	human	NZ	✓	
SRR14717099	10/02/2021	-	human	NZ	✓	
SRR14717101	10/02/2021	-	human	NZ	✓	
SRR14716940	11/02/2021	-	human	NZ	✓	
SRR14716941	11/02/2021	-	human	NZ	✓	
SRR14717098	14/02/2021	Yes	human	NZ	✓	
SRR14717100	14/02/2021	-	human	NZ	✓	
SRR14717094	15/02/2021	-	human	NZ	✓	
SRR14717097	15/02/2021	Yes	human	NZ	✓	
SRR14902941	15/02/2021	-	human	NZ	✓	
SRR14717095	16/02/2021	-	human	NZ	✓	
SRR14717089	18/02/2021	Yes	human	NZ	✓	✓
SRR14717074	3/03/2021	-	human	NZ	✓	
SRR14717057	9/03/2021	-	human	NZ	✓	
SRR14717053	10/03/2021	-	human	NZ	✓	
SRR14717051	12/03/2021	-	human	NZ	✓	
SRR14717017	25/03/2021	-	human	NZ	✓	✓
SRR14717001	6/04/2021	-	human	NZ	✓	
SRR14717000	7/04/2021	-	human	NZ	✓	
SRR14717008	7/04/2021	-	human	NZ	✓	
SRR35900399	7/04/2021	-	human	NZ	✓	
SRR14716890	11/04/2021	-	human	NZ	✓	
SRR14902916	29/04/2021	-	human	NZ	✓	
SRR14902915	30/04/2021	-	human	NZ	✓	
SRR14902902	4/05/2021	-	human	NZ	✓	
SRR14902889	14/05/2021	-	human	NZ	✓	
SRR14902882	18/05/2021	-	human	NZ	✓	✓
SRR35900397	9/06/2021	-	human	NZ	✓	
SRR35900398	12/06/2021	-	human	NZ	✓	
SRR35900396	14/06/2021	-	human	NZ	✓	
SRR35900395	21/06/2021	-	human	NZ	✓	
SRR35900394	26/06/2021	-	human	NZ	✓	
SRR35900393	11/07/2021	-	human	NZ	✓	
SRR35900392	22/07/2021	-	human	NZ	✓	
SRR35900390	8/09/2021	-	human	NZ	✓	✓
SRR35900389	4/11/2021	-	human	NZ	✓	
SRR35900388	27/11/2021	-	human	NZ	✓	
SRR35900387	9/12/2021	-	human	NZ	✓	
SRR35900386	12/12/2021	-	human	NZ	✓	

SRA accession	Collection date	2021 NZ outbreak	Host	Country	coreSNP analysis	Temporal phylogenetic reconstruction
SRR35900385	22/12/2021	-	human	NZ	✓	
SRR35900384	28/12/2021	-	human	NZ	✓	
SRR35900381	4/01/2022	-	human	NZ	✓	✓
SRR35900383	4/01/2022	-	human	NZ	✓	
SRR35900382	5/01/2022	-	human	NZ	✓	
SRR35900379	11/01/2022	-	human	NZ	✓	
SRR35900378	20/01/2022	-	human	NZ	✓	
SRR35900419	27/01/2022	-	human	NZ	✓	
SRR35900425	3/02/2022	-	human	NZ	✓	
SRR35900424	4/02/2022	-	human	NZ	✓	✓
SRR35900422	8/02/2022	-	human	NZ	✓	
SRR35900423	8/02/2022	-	human	NZ	✓	
SRR35900421	14/02/2022	-	human	NZ	✓	
SRR35900420	15/02/2022	-	human	NZ	✓	✓
SRR35900418	1/03/2022	-	human	NZ	✓	
SRR35900416	11/03/2022	-	human	NZ	✓	
SRR35900415	20/03/2022	-	human	NZ	✓	
SRR35900414	30/03/2022	-	human	NZ	✓	
SRR35900411	2/04/2022	-	human	NZ	✓	
SRR35900413	4/04/2022	-	human	NZ	✓	
SRR35900412	7/04/2022	-	human	NZ	✓	✓
SRR35900409	12/04/2022	-	human	NZ	✓	
SRR35900410	12/04/2022	-	human	NZ	✓	
SRR35900408	15/04/2022	-	human	NZ	✓	
SRR35900407	24/04/2022	-	human	NZ	✓	
SRR35900404	26/04/2022	-	human	NZ	✓	
SRR35900405	26/04/2022	-	human	NZ	✓	
SRR35900402	29/04/2022	-	human	NZ	✓	
SRR35900403	29/04/2022	-	human	NZ	✓	
SRR35900377	12/05/2022	-	human	NZ	✓	✓
SRR35900375	23/05/2022	-	human	NZ	✓	✓
SRR35900376	26/05/2022	-	human	NZ	✓	
SRR35901115	23/04/2021	-	sheep	NZ	✓	✓

*Estimated collection date; SRA - Sequence Read Archive.

Table S2. New Zealand *Salmonella* Enteritidis notified human cases stratified by multi-locus sequence type (MLST*)*, 2020-2023.

MLST	Count
ST11	193
ST183	160
ST180	6
ST1925	4
ST1975	1
ST3233	1
ST3347	1
ST74	1
Total	367

Table S3. New Zealand *Salmonella* Enteritidis ST183 and non-ST183 notified human cases by age group, 2020-2023.

		ST183				Non ST183			
Age category (years)	Person years at risk	Cases	Rate [95% CI]	Rate ratio (95%CI)	P-value	Cases	Rate [95% CI]	Rate ratio (95% CI)	P-value
0 to 4	1153548	22	19.1 [12, 28.9]	7.7 [3.4, 19.8]	0.0000002	32	27.7 [19, 39.2]	5 [2.8, 9.3]	0.0000001
5 to 9	1246944	9	7.2 [3.3, 13.7]	3 [1.1, 8.4]	0.035	18	14.4 [8.6, 22.8]	2.6 [1.3, 5.2]	0.006
10 to 14	1344696	5	3.7 [1.2, 8.7]	1.5 [0.4, 4.9]	0.476	11	8.2 [4.1, 14.6]	1.5 [0.7, 3.2]	0.330
15 to 19	1282548	6	4.7 [1.7, 10.2]	1.9 [0.6, 5.9]	0.254	7	5.5 [2.2, 11.2]	1 [0.4, 2.3]	0.991
20 to 29	2590668	18	6.9 [4.1, 11]	2.8 [1.2, 7.3]	0.015	20	7.7 [4.7, 11.9]	1.4 [0.7, 2.7]	0.332
30 to 39	2878464	7	2.4 [1, 5]	1 (ref)		16	5.6 [3.2, 9.0]	1 (ref)	
40 to 49	2471940	14	5.7 [3.1, 9.5]	2.3 [0.9, 6.2]	0.066	21	8.5 [5.3, 13]	1.5 [0.8, 3.0]	0.204
50 to 59	2506836	19	7.6 [4.6, 11.8]	3.1 [1.3, 8]	0.007	32	12.8 [8.7, 18]	2.3 [1.3, 4.3]	0.006
60+	4500012	60	13.3 [10.2, 17.2]	5.4 [2.6, 13]	0.0000002	50	11.1 [8.2, 14.6]	2 [1.2, 3.6]	0.012
0 to 4 vs 5+				2.6 [1.6, 4]	0.00020			3 [2.0, 4.3]	0.000001
60+ vs 0-59				**2.1 [1.5, 2.8]**	**0.00002**			1.1 [0.8, 1.5]	0.570

Table S4. New Zealand urban rural profile of *Salmonella* Enteritidis ST183 and non-ST183 notified human cases, 2020-2023.

		ST183				Non ST183			
Age category (years)	Person years at risk	Cases	Rate [95% CI]	Rate ratio (95%CI)	P-value	Cases	Rate [95% CI]	Rate ratio (95% CI)	P-value
Rural	3242000	43	13.3 [9.6, 17.9]	**2.3 [1.6, 3.2]**	**0.00002**	30	9.3 [6.2, 13.2]	1 [0.7, 1.5]	1.00
Urban	17556800	102	5.8 [4.7, 7.1]	(ref)		163	9.3 [7.9, 10.8]	(ref)	

Table S5. New Zealand *Salmonella* Enteritidis ST183 exposures compared to non-ST183 notified human cases, 2020-2023.

Exposure	ST183 cases exposed	ST183 cases not exposed	% ST183 cases exposed	Non-ST183 cases exposed	Non-ST183 cases not exposed	% Non-ST183 cases exposed	Odds ratio* (95%CI)	p-value (Fisher exact)
Farm animals	33	90	26.8	19	117	14.0	**2.3 [1.2, 4.5]**	**0.01**
Swimming in pool	6	154	3.8	5	202	2.4	1.6 [0.4, 6.6]	0.54
Swimming in streams, river, sea	8	152	5.0	8	199	3.9	1.3 [0.4, 4.1]	0.62
Contact with sick animals	3	115	2.5	3	127	2.3	1.1 [0.1, 8.4]	1.00
Food premises	54	63	46.2	67	77	46.5	1.0 [0.6, 1.7]	1.00
Untreated water	19	93	17.0	21	98	17.6	1.0 [0.5, 2.0]	1.00
Contact with human faeces	10	111	8.3	12	126	8.7	0.9 [0.4, 2.5]	1.00
Contact with other symptomatic people	9	110	7.6	18	133	11.9	0.6 [0.2, 1.5]	0.31
Attended school	15	119	11.2	29	127	18.6	0.6 [0.3, 1.1]	0.10
Recreational contact with water	15	108	12.2	33	107	23.6	**0.5 [0.2, 0.9]**	**0.02**
International travel	0	137	0.0	57	125	31.3	**<0.1 [0.0, 0.1]**	**<0.000001**

‘Recreational contact with water’ is a parent question for ‘Swimming in pool’ and ‘Swimming in streams, river, sea.

*Odds of exposure for ST183 cases divided by odds of exposure for non-ST183 cases.

Table S6. Phenotypic anti-microbial resistance results for randomly selected NZ ST183 human isolates included in this study.

	16ER0960	22ER0025	22ER0141	22ER0564	22ER0767	22ER1032
Amoxicillin-clavulanic acid	S	S	S	S	S	S
Ampicillin	S	S	S	S	S	S
Azithromycin	S	S	S	S	S	S
Chloramphenicol	S	S	S	S	S	S
Co-trimoxazole	S	S	S	S	S	S
Cefotaxime	S	S	S	S	S	S
Ceftazidime	S	S	S	S	S	S
Gentamicin	S	S	S	S	S	S
Meropenem	S	S	S	S	S	S
Pefloxacin	S	S	S	S	S	S
Tetracycline	S	S	S	S	S	S

S = susceptible
